# *In silico* detection and characterization of novel virulence proteins of the emerging poultry pathogen *Gallibacterium anatis*

**DOI:** 10.5808/gi.22006

**Published:** 2022-12-30

**Authors:** L. G. T. G Rajapaksha, C. W. R Gunasekara, P. S. de Alwis

**Affiliations:** 1 Veterinary Medical Center and College of Veterinary Medicine, Jeonbuk National University, Jeonju 54596, Korea; 2 College of Fisheries Science, Pukyong National University, Busan 48513, Korea

**Keywords:** bioinformatics, hypothetical proteins, *in silico* detection, metabolic pathways, virulence, 3D structure

## Abstract

The pathogen *Gallibacterium anatis* has caused heavy economic losses for commercial poultry farms around the world. However, despite its importance, the functions of its hypothetical proteins (HPs) have been poorly characterized. The present study analyzed the functions and structures of HPs obtained from *Gallibacterium anatis* (NCTC11413) using various bioinformatics tools. Initially, all the functions of HPs were predicted using the VICMpred tool, and the physicochemical properties of the identified virulence proteins were then analyzed using Expasy's ProtParam server. A virulence protein (WP_013745346.1) that can act as a potential drug target was further analyzed for its secondary structure, followed by homology modeling and three-dimensional (3D) structure determination using the Swiss-Model and Phyre2 servers. The quality assessment and validation of the 3D model were conducted using ERRAT, Verify3D, and PROCHECK programs. The functional and phylogenetic analysis was conducted using ProFunc, STRING, KEGG servers, and MEGA software. The bioinformatics analysis revealed 201 HPs related to cellular processes (n = 119), metabolism (n = 61), virulence (n = 11), and information/storage molecules (n = 10). Among the virulence proteins, three were detected as drug targets and six as vaccine targets. The characterized virulence protein WP_013745346.1 is proven to be stable, a drug target, and an enzyme related to the citrate cycle in the present pathogen. This enzyme was also found to facilitate other metabolic pathways, the biosynthesis of secondary metabolites, and the biosynthesis of amino acids.

## Introduction

*Gallibacterium anatis* (earlier known as *Pasteurella anatis*), which belongs to the family Pasteurellaceae, is an emerging disease-causing organism in poultry [1]. This bacterium has been isolated from various animals including chickens, turkeys, geese, ducks, pheasants, partridges, budgerigars, peacocks, cage birds, and wild birds [1-3]. There have been debates about whether this bacterium is pathogenic or nonpathogenic since it is found as a common part of the microbiota in upper respiratory tract and lower reproductive tract of healthy chickens [2,4,5]. However, increasing evidence indicates that *G. anatis* is associated with a wide range of pathological changes, leading to decreased egg production and lowered animal welfare in commercial poultry farms [6,7]. The disease is most likely to occur in intensively reared poultry farms (such as those raising broiler chickens) and incurs a high rate of mortality unless treated [1]. Therefore, determining the virulence factors of *G. anatis* would play an important role in proposing better control and prevention methods.

In recent years, numerous genomes have been sequenced with the help of next-generation sequencing technology and are available in public databases, such as that operated by the National Center for Biotechnology Information (NCBI) [8]. As a result, genomes with hypothetical proteins (HPs) have been deposited in sequence databases instead of experimentally confirmed facts due to their functional importance. Moreover, in some circumstances, because of limitations in illustrations (experimental validation techniques), expenditures, and the time required for the corresponding methodologies, whole-genome annotations have not been archived. Furthermore, the large amount of HPs in a genome naturally creates difficulties in data analysis [8]. This encourages *in silico* analysis, which utilizes and produces experimental information on HPs [9]. Establishing a structural and functional annotation for HPs may also play a significant role in elucidating protein pathways and cascades, helping to complete the currently approximate records on a variety of proteins [8,10]. Bioinformatics methods using discriminative algorithms and databases are the best approach to influence laboratory-based experimental procedures. Since these algorithms produce precise experimental results, they can be used to complete the functional and structural annotation of HPs [8].

The present study employed an *in silico* approach and predicted the functions of all HPs in the *G. anatis* reference genome (NCTC11413). Following identification, the physicochemical properties of the virulence-associated HPs were examined. Among them, a virulence protein within the pH range of 6–7 was identified as a drug target and analyzed for secondary structures, leading to the production of its first three-dimensional (3D) model by the *ab initio* method, and finally enabling the completion of its functional annotation. We believe that the present study provides a convenient methodology to analyze HPs and their functions in prokaryotic genomes.

## Methods

### Sequence retrieval and selection of HPs

The reference genome NCTC11413 was retrieved from the NCBI database. We observed that the genome consisted of 2,404 coding sequences. Upon analysis (https://www.ncbi.nlm.nih.gov/assembly/GCF_900450735.1/), 201 HPs were identified. All 201 HPs were separated from the genome using Geneious Prime version 2020.0.5.

### *In silico* prediction of virulence factors, cellular processes, information/storage, and metabolism molecules

Identifying the functions of HPs plays a vital role in understanding a bacterium’s metabolic pathways and pathogenesis. The VICMpred tool (http://crdd.osdd.net/raghava/vicmpred/index.html) was employed for the identification of possible virulence factors, cellular processes, information/storage, and metabolism molecules among HPs from the reference strain NCTC11413. The VICMpred server is a support vector machine (SVM)–based method with the amino acid and dipeptide composition patterns of bacterial protein sequences [11]. The server provides an overall detection accuracy of 70.75%. At the end of the selection process, all virulence-associated HPs were selected for further characterization.

### Physicochemical properties of virulence proteins

The physicochemical parameters of all virulence proteins were studied using Expasy's ProtParam server (https://web.expasy.org/protparam/), which was then used for computed theoretical measurements such as molecular weight, extinction coefficient, instability index, aliphatic index, and grand average of hydropathicity (GRAVY). The extinction coefficient measures the amount of light that a protein can absorb at a certain wavelength. The instability index provides an estimation of the stability of a protein in a test tube, with a value of 40 indicating instability. The aliphatic index of a protein is described as the relative volume occupied by aliphatic side-chain amino acids. The GRAVY value for a peptide and/or protein is calculated as the sum of the hydropathy values of all of the amino acids, divided by the number of residues in the query sequence [12].

### Subcellular localization and protein classification

It is a well-known fact that proteins present in the cytoplasm can serve as possible drug targets, while membrane proteins found on the surface are considered as vaccine targets. Thus, the subcellular localization of virulence proteins was predicted using the PSLpred online web server [it is a hybrid approach-based method that integrates PSI-BLAST and three SVM modules based on compositions of residues, dipeptides, and physicochemical properties] (http://crdd.osdd.net/raghava/pslpred/) and PSORT (a computer program for the prediction of protein localization sites in cells) (https://www.psort.org/). Moreover, the SignalP server (http://www.cbs.dtu.dk/services/SignalP/) was used to determine the presence of transmembrane helices and signal peptides [13-15]. This information is important for determining whether a protein is a membrane protein, secretory protein, or cytoplasmic protein. Following this, a virulence protein within the pH range of 6–7 was subjected to further characterization.

### Functional domains, interaction network, and phylogenetic relationships of the protein

A virulence protein (WP_013745346.1) was examined to identify its precise functional domains using Pfam [16], HmmScan [17], Scanprosite [18], InterProScan [19], and SMART [20]. Additionally, the ProFunc server and STRING database were employed to understand possible functional interactions associated with the virulence protein [21,22]. Upon analysis, a phylogenetic tree was constructed using 14 other reference protein sequences in mega software version 7 [23] and the potential metabolic pathways were assessed using the Kyoto Encyclopedia of Genes and Genomes [24].

### Secondary structure analysis

The SOPMA server (https://npsa-prabi.ibcp.fr/NPSA/npsa_sopma.html) was used to predict the secondary structure (helix, sheets, and coils) of the virulence protein WP_013745346.1 [25]. In addition, the PSIPRED server (http://bioinf.cs.ucl.ac.uk/psipred/) was utilized to confirm the results achieved from SOPMA [26].

### Homology modeling of the HP

The possible 3D structure of the virulence protein (WP_013745346.1) was created by an alignment approach on the SWISS-MODEL protein structure homology modeling server (https://swissmodel.expasy.org/) and the Phyre2 server [12,25].

### Quality assessment of the 3D model and visualization

The early structural model of the achieved protein was checked for mistakes in the 3D structure using the ERRAT and Verify3D programs (https://servicesn.mbi.ucla.edu/) for structural examination and confirmation of protein modeling [26,27]. Finally, the PROCHECK structural evaluation server was used to assess the quality of the 3D structure [28]. The visualization of creating the model was accomplished by Geneious Prime version 2020.0.5.

### Submission of the model to a protein model database

The protein model was created and successfully submitted to the Protein Model Database for the virulence protein (WP_013745346.1).

## Results and Discussion

### *In silico* prediction of virulence factors, cellular processes, information/storage, and metabolism molecules

Initially, by using the reference genome NCTC11413 we identified 201 uncharacterized HPs for *G. anatis*. These 201 HPs were analyzed using the VICMpred tool to understand their functional attributes. In return, the HPs were divided into four groups: 119 for cellular processes, 61 for metabolism molecules, 11 for virulence, and 10 for information/storage. The 11 virulence proteins identified in the present study were subjected to further characterization (Supplementary Tables 1 and 2).

### Physicochemical properties, subcellular localization, and protein classification of the virulence proteins

The novel virulence proteins identified had 333–3,033 nucleotides and 111–1,011 amino acids (Table 1). Among the virulence proteins, the highest extinction coefficient (1.4 × 10^5^) was observed in the HP WP_043885272.1. The instability index indicated that WP_013745190.1, WP_013746187.1, and WP_013746977.1 were unstable proteins, whereas the others were stable proteins (Table 2). Following this, the prediction of subcellular localization using PSORT indicated that 10 virulence proteins were cytoplasmic membrane proteins, whereas PSLpred showed three cytoplasmic proteins, two extracellular proteins, and six outer or inner membrane proteins. It is well known that proteins in the cytoplasm are possible drug targets, while membrane proteins are considered to be vaccine targets [29]. Cytoplasmic proteins play a crucial role in metabolic pathways that are critical to the survival of the pathogen inside the host organism [30]. Therefore, the three proteins detected in the cytoplasm (from both PSLpred and PSORT servers) can be used as drug targets, whereas the six membrane proteins could be used as vaccine targets against the present pathogen (Table 3). Moreover, the virulence-associated HP named WP_013746977.1 was identified as a signal peptide, whereas WP_013745329.1 was identified as a lipoprotein signal peptide. The protein structure of WP_013745346.1 (protein in the pH range of 6–7 and a possible drug target).

### Functional annotation of the virulence protein WP_013745346.1

**Fig. 1.**
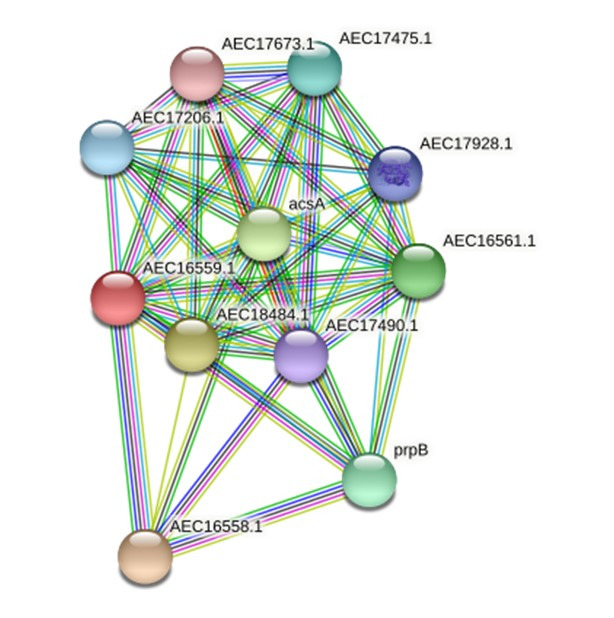
A STRING database search was carried out to identify a possible functional interaction network of the virulence protein WP_013745346.1 (AEC16559.1). The identified functional protein partners with the corresponding scores were as follows: AEC16558.1 (0.997), AEC18484.1 (0.966), acsA (0.962), AEC16561.1 (0.952), prpB (0.948), AEC17475.1 (0.920), AEC17206.1 (0.908), AEC17928.1 (0.907), AEC17490.1(0.877), and AEC17673.1 (0.841).

All five employed tools indicated that the virulence protein WP_013745346.1 is an enzyme (known as methylcitrate synthase) primarily associated with the citrate cycle (Supplementary Table 3). ProFunc analysis indicated it is related to both biological processes (cellular process [74.47], cellular metabolic process [74.47], metabolic process [74.47], cofactor metabolic process [56.61]) and biochemical processes (catalytic activity [74.47], transferase activity [73.74], transferase activity/transferring acyl groups [47.82], transferase activity/ transferring acyl groups/acyl groups converted into alkyl on transfer [41.91]). The STRING protein network analysis suggested that the virulence protein WP_013745346.1 is associated with other functional proteins such as acsA and prpB (Fig. 1). The phylogenetic analysis with other reference sequences revealed that WP_013745346.1 is similar to other bacterial methylcitrate synthases (Supplementary Fig. 1). Moreover, this virulence protein was related to the biosynthesis of secondary metabolites, microbial metabolism in diverse environments, carbon metabolism, the biosynthesis of amino acids, and glyoxylate and dicarboxylate metabolism (Supplementary Fig. 2). This enzyme is a key determinant of propanoate degradation in micro-organisms [31]. Previous studies also suggested that the presence of methylcitrate synthase, malate synthase, and methylcitrate dehydrate is essential to the intercellular growth, metabolism, and virulence of bacteria, such as *Mycobacterium tuberculosis* [32]. Moreover, methylcitrate synthase in fungi, such as *Aspergillus fumigatus*, has the potential to detoxify propionyl-CoA, which is a byproduct of protein utilization; therefore, it can be used as a novel drug target [33].

### Secondary structure of the virulence protein WP_013745346.1

**Fig. 2.**
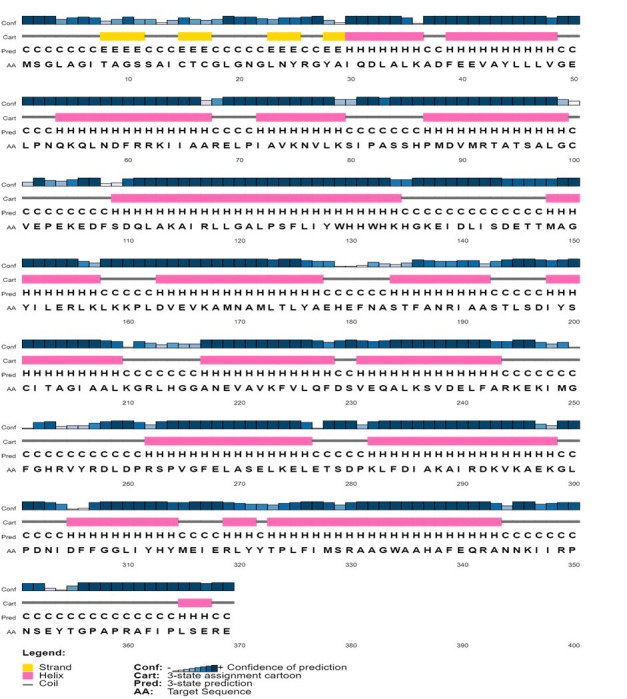
The secondary structure of the virulence protein WP_013745346.1 was predicted using the PSIPRED server.

The secondary structure of the protein WP_013745346.1 was predicted using the SOPMA server. Alpha helices were found to be the most predominant (53.93%), followed by random coils (32.25%) and extended strands (8.40%). Beta-turns accounted for 5.42% of the structure of this protein. The predicted secondary structure for the virulence protein WP_013745346.1 from the PSIPRED server was also similar (Fig. 2).

### Homology modeling of the virulence protein WP_013745346.1

**Fig. 3.**
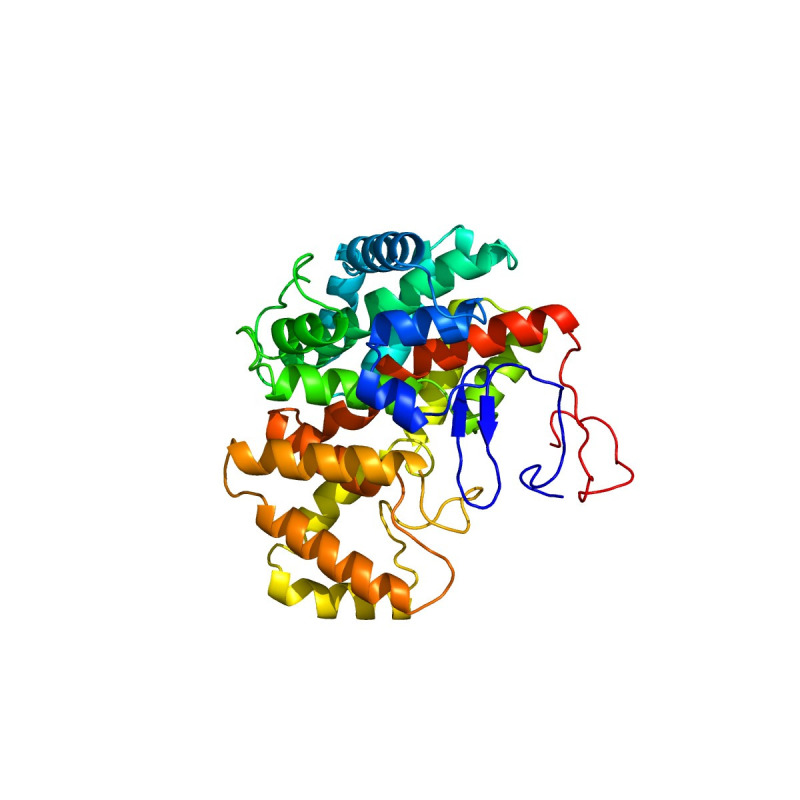
Three-dimensional structural analysis and visualization of the virulence protein WP_013745346.1 with Geneious Prime version 2020.0.5.

*G. anatis* infections have been reported in recent years at intensively reared poultry farms. However, its virulence-related factors have not been fully elucidated so far. Previous studies have demonstrated that the identification of virulence proteins from HPs plays a significant role in understanding its pathogenesis [28,29]. *In silico* analysis can help determine the biological functions of virulence proteins [8]. These predictions can be further strengthened by determining the 3D structures of virulence proteins using homology modeling. Homology modeling identifies the 3D structure of a selected protein sequence through alignment to one or more proteins of other known structures [34]. To perform homology modeling, the query sequence of virulence protein WP_013745346.1 was submitted to the SWISS-MODEL server. The server performed a BLASTP search for the respective protein sequence to identify templates associated with homology modeling. The highest identity of 36% observed for this virulence protein indicated that WP_013745346.1 is novel and no similar template structure is currently present in other databases. Following this, we determined the 3D structure of the virulence protein WP_013745346.1 by the *ab initio* method through the Phyre2 server, which gave 99.8% confidence in the model (Fig. 3). A comparative analysis of *C. burnetii* and *M. tuberculosis* methylcitrate synthases to WP_013745346.1, showed a common structural domain (citrate synthase, C-terminal domain), cellular location (cytoplasm), and molecular functions (Supplementary Fig. 3).

### Quality assessment and visualization

**Fig. 4.**
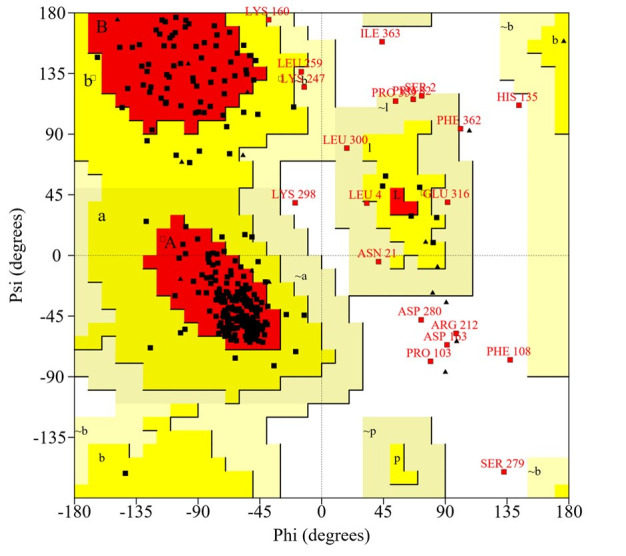
Ramachandran plot of the modeled structure for the virulence protein WP_013745346.1 validated by the PROCHECK program.

The reliability of the created protein model was assessed using the ERRAT server, which analyzes the statistics of non-bonding interactions between diverse atom types, based on characteristic atomic interactions. The overall quality factor was found as 96.096%, which was sufficient to use this model. As shown by the Verify3D program, the results indicated 86.60% of residues had an average 3D (atomic model) – 1D (amino acid) score ≥0.2, meaning that this structure was compatible and genuinely good. Next, validation through a Ramachandran plot analysis showed that the distribution of φ and ψ angles in the model were within the limits (Table 4), 90.2% of the residues are in the most favored region of the plot, indicating that the model was valid (Fig. 4).

### Data availability

The model created for the virulence protein WP_013745346.1 is currently available in the protein model database under reference number-PM0083267.

### Summary

The present study aimed to characterize the HP functions of the emerging poultry pathogen *G. anatis*, as well as to create the first 3D structure and propose possible functions of the virulence protein WP_013745346.1. We observed that this novel protein is a stable cytoplasmic protein and functions as an enzyme in the citrate cycle. This protein was observed to be central to several other metabolic pathways. Therefore, the novel virulence protein studied here may have a significant impact on the pathogenesis of *G. anatis*.

## Figures and Tables

**Table 1.** Novel virulence proteins identified in the *Gallibacterium anatis* NCTC11413 genome by the VCMPred online server

Protein name	Sequence length (bp)	Protein length (AA)	VICM prediction	Value
WP_013745190.1	333	111	Virulence factor	0.77
WP_013745886.1	492	164	Virulence factor	0.78
WP_013745329.1	519	173	Virulence factor	0.83
WP_013746187.1	555	185	Virulence factor	2.92
WP_013745598.1	711	237	Virulence factor	1.05
WP_013747269.1	753	251	Virulence factor	0.45
WP_013745861.1	831	277	Virulence factor	0.73
WP_013745346.1	1,110	370	Virulence factor	1.22
WP_013746977.1	1,116	372	Virulence factor	1.03
WP_013746705.1	1,260	420	Virulence factor	2.53
WP_043885272.1	3,033	1,011	Virulence factor	1.15

**Table 2.** Physicochemical properties of 11 virulence proteins of *Gallibacterium anatis* with the ProtParam online server

		Molecular weight (Da)	Extinction coefficient	Instability index	Aliphatic index	Grand average of hydropathicity	Theoretical pH	Stability
WP_013745190.1	Virulence	13,148.94	25,900	56.76	100.09	–0.385	4.99	Unstable
WP_013745886.1	Virulence	17,489	18,450	26.69	81.23	–0.45	4.61	Stable
WP_013745329.1	Virulence	17,394.37	17,200	13.08	70.29	–0.473	4.93	Stable
WP_013746187.1	Virulence	20,970.83	13,410	84.02	91.68	–0.624	6.92	Unstable
WP_013745598.1	Virulence	27,340.22	27,975	30.81	86.82	–0.383	8.2	Stable
WP_013747269.1	Virulence	27,300	21,430	31.27	90.6	–0.244	9.54	Stable
WP_013745861.1	Virulence	31,472	37,360	30.65	83.01	–0.386	5.37	Stable
WP_013745346.1^a^	Virulence	41,052	36,120^a^	31.37	95.04	–0.118	6.57	Stable
WP_013746977.1	Virulence	41,061	24,200	41.25	61.73	–0.814	4.88	Unstable
WP_013746705.1	Virulence	45,399	39,310	16.98	89.19	–0.284	5.55	Stable
WP_043885272.1	Virulence	108,467	149,910	21.7	66.4	–0.698	4.55	Stable

a Newly characterized proteins in the present study are indicated.

**Table 3.** Subcellular localization and classification of 11 virulence proteins of *Gallibacterium anatis* with PSORT, PSLpred, and SignalP-5.0 online servers. (unknown: the PSORT server predicted the same values for all the functional categories)

Protein	Protein length (AA)	PSORT			PSL Pred (Property-based)		SignalP-5.0			
		Localization	Value	Internal helices	Value	Subcellular localization	Signal peptide	TAT signal peptide	Lipoprotein signal peptide	Other
WP_013745190.1	111	Cytoplasmic membrane	9.5	8	–0.11	Cytoplasmic protein^a^	0.0032	0.001	0.0007	0.9951
WP_013745886.1	164	Cytoplasmic membrane	9.5	16	0.43	Extracellular protein	0.0135	0.0007	0.0036	0.9822
WP_013745329.1	173	Cytoplasmic membrane	9.5	18	–0.54	Inner membrane protein^b^	0.0012	0.0001	0.9985	0.0002
WP_013746187.1	185	Unknown	Inconclusive	Inconclusive	–0.47	Outer membrane protein^b^	0.0267	0.0034	0.0083	0.9616
WP_013745598.1	237	Cytoplasmic membrane	9.5	18	–0.07	Cytoplasmic protein^a^	0.0074	0.0001	0.991	0.0015
WP_013747269.1	251	Cytoplasmic membrane	9.5	15	0.54	Outer membrane protein^b^	0.0182	0.0053	0.0034	0.9731
WP_013745861.1	277	Cytoplasmic membrane	9.5	8	1.10	Outer membrane protein^b^	0.0095	0.0049	0.0021	0.9835
WP_013745346.1	370	Cytoplasmic membrane	9.46	4	1.43	Cytoplasmic protein^a^	0.0749	0.0013	0.33	0.5938
WP_013746977.1	372	Cytoplasmic membrane	9.46	27	–0.48	Extracellular protein	0.9814	0.0001	0.018	0.0004
WP_013746705.1	420	Cytoplasmic membrane	9.5	15	1.07	Outer membrane protein^b^	0.0077	0.0012	0.001	0.9901
WP_043885272.1	1011	Cytoplasmic membrane	9.46	55	–0.18	Outer membrane protein^b^	0.1173	0.065	0.0057	0.8119

a Possible drug targets are indicated.

b Vaccine targets are indicated.

**Table 4.** Ramachandran plot statistics of the virulence protein WP_013745346.1 predicted by the PROCHECK server

Ramachandran plot statistics	No. (%)
Residues in the most favored regions [A, B, L]	295 (90.2)
Residues in the additional allowed regions [a, b, l, p]	15 (4.6)
Residues in the generously allowed regions [a, b, l, p]	8 (2.4)
Residues in the disallowed regions	9 (2.8)
No. of non-glycine and non-proline residues	327
No. of end-residues (excl. Gly and Pro)	2
No. of glycine residues (shown in triangles)	24
No. of proline residues	16
Total no. of residues	369

## Supplementary Materials

Supplementary Table. 1. Functional characterization of 201 hypothetical proteins of the poultry pathogen *Gallibacterium anatis*

Supplementary Table. 2. Novel proteins identified from poultry pathogen Gallibacterium anatis associated with virulence, metabolism, Information and cellurer processes.

Supplementary Table. 3. Functional domain prediction by five web servers for the virulence protein WP_013745346.1

Supplementary Fig. 1. Phylogenetic relationships of the virulence protein WP_013745346.1 with other reference protein sequences. (The evolutionary history was inferred using the neighbor-joining method. The bootstrap tree was inferred from 1,000 replicates and taken to represent the evolutionary history. The evolutionary distances were computed using the Poisson correction method and are in the units of the number of amino acid substitutions per site. The analysis involved 15 amino acid sequences. There was a total of 262 positions in the final dataset. The evolutionary analysis was conducted in MEGA7).

Supplementary Fig. 2. Potential metabolic pathways associated with the virulence protein WP_013745346.1 by KAAS (KEGG automatic annotation server analysis).

Supplementary Fig. 3. Comparative analysis of the 3D structure, structural domains, cellular location, and molecular functions of reference methylcitrate synthases to WP_013745346.1
