# Supplementary material for: In silico detection and characterization of novel virulence proteins of the emerging poultry pathogen Gallibacterium anatis
Source: Genomics Inform. 2022 Dec 30;20(4):e41. doi: 10.5808/gi.22006 (PMC9847380; doi:10.5808/gi.22006)
Supplement: Supplementary Table. 1. — Functional characterization of 201 hypothetical proteins of the poultry pathogen Gallibacterium anatis [file gi-22006suppl1.pdf]

Supplementary Table 1. Functional characterization of 201 hypothetical proteins of the poultry pathogen *Gallibacterium anatis*

Protein	Sequence length	Protein length	VICM prediction	Value
WP_080558816.1	90		Cellular process	1.12
WP_013747009.1	123	40	Cellular process	0.99
WP_080558812.1	132	42	Cellular process	1.06
WP_013746397.1	150	50	Cellular process	0.98
WP_158305795.1	150	49	Cellular process	1.45
WP_021460868.1	165	54	Metabolism molecule	1.64
WP_013747279.1	180	59	Cellular process	0.98
WP_013745256.1	180	59	Cellular process	1.29
WP_013745173.1	180	59	Cellular process	0.99
WP_013746093.1	183	60	Cellular process	1.12
WP_013746093.2	183	60	Cellular process	2.12
WP_013745012.1	183	61	Metabolism molecule	1.52
WP_013745153.1	186	62	Cellular process	1.12
WP_013745024.1	186	62	Metabolism molecule	1.52
WP_080558809.1	189	62	Metabolism molecule	1.2
WP_158305796.1	189	62	Metabolism molecule	0.89
WP_013745462.1	189	62	Cellular process	1.12
WP_013745018.1	189	63	Metabolism molecule	1.03
WP_013747125.1	192	63	Information and storage	1.2
WP_013746716.1	192	63	Information and storage	1.01
WP_009116473.1	195	65	Cellular process	0.99
WP_013747116.1	198	66	Information and storage	0.74
WP_013746417.1	198	66	Information and storage	0.79
WP_013747223.1	204	67	Metabolism molecule	0.91
WP_013745169.1	204	67	Cellular process	0.87
WP_148234063.1	207	69	Metabolism molecule	1.2
WP_013745242.1	207	69	Metabolism molecule	0.99
WP_013745193.1	210	69	Metabolism molecule	0.66
WP_013747168.1	213	70	Metabolism molecule	2.06
WP_013747118.1	216	71	Cellular process	0.98
WP_013747241.1	222	73	Cellular process	0.87
WP_013746407.1	222	73	Cellular process	1.29
WP_013747272.1	225	74	Cellular process	0.69
WP_013747256.1	225	74	Cellular process	0.75
WP_013747153.1	225	74	Cellular process	1.09
WP_013745488.1	225	74	Metabolism molecule	3.022
WP_013745622.1	228	76	Cellular process	1.9
WP_013745436.1	228	76	Metabolism molecule	1.19
WP_013747117.1	231	77	Cellular process	0.67

WP_148234062.1	237	78	Cellular process	0.99
WP_013746388.1	237	78	Cellular process	0.67
WP_013745177.1	246	82	Cellular process	2.02
WP_013745177.1	246	82	Cellular process	2
WP_013746433.1	246	82	Metabolism molecule	1.23
WP_013747210.1	246	82	Metabolism molecule	0.93
WP_013745014.1	249	83	Cellular process	1.16
WP_013747172.1	249	83	Cellular process	2.06
WP_013745155.1	252	84	Cellular process	1.84
WP_158305800.1	252	84	Cellular process	1.23
WP_013746788.1	255	84	Cellular process	1.43
WP_013747066.1	255	84	Metabolism molecule	1.11
WP_013747171.1	255	85	Cellular process	1.56
WP_013745011.1	258	86	Cellular process	1.76
WP_013745015.1	261	87	Metabolism molecule	1.22
WP_013745197.1	261	87	Metabolism molecule	0.94
WP_013747126.1	264	88	Cellular process	1.23
WP_013745601.1	267	89	Metabolism molecule	1.89
WP_013745045.1	270	90	Cellular process	1.23
WP_013745013.1	273	91	Cellular process	1.56
WP_013745035.1	273	91	Metabolism molecule	1.88
WP_013745541.1	273	91	Cellular process	1.35
WP_013747190.1	273	91	Cellular process	0.79
WP_013747284.1	273	91	Cellular process	1.43
WP_013745144.1	279	93	Metabolism molecule	1.28
WP_158305802.1	279	93	Cellular process	1.23
WP_013745047.1	282	94	Cellular process	1.78
WP_013747280.1	282	94	Cellular process	0.78
WP_013747188.1	285	95	Cellular process	1.021
WP_013745732.1	288	96	Cellular process	1.03
WP_013746685.1	288	96	Cellular process	1.68
WP_013745838.1	294	98	Cellular process	1
WP_013747180.1	294	98	Cellular process	1.23
WP_013747275.1	294	98	Metabolism molecule	2.43
WP_013746402.1	297	99	Cellular process	1.18
WP_013747283.1	297	99	Cellular process	0.77
WP_013745157.1	306	102	Metabolism molecule	1.88
WP_013745187.1	306	102	Cellular process	1.12
WP_013746386.1	306	102	Metabolism molecule	1.34
WP_013746411.1	306	102	Cellular process	1.57
WP_013744895.1	318	106	Cellular process	1.54
WP_013745191.1	321	107	Cellular process	0.67
WP_013745257.1	321	107	Cellular process	0.93

WP_013747127.1	321	107	Metabolism molecule	1.84
WP_013747212.1	327	109	Metabolism molecule	1.11
WP_013745192.1	330	110	Cellular process	1.34
WP_013745190.1	333	111	Virulence factor	0.77
WP_013745054.1	336	112	Cellular process	1.88
WP_013746603.1	336	112	Cellular process	0.88
WP_013746701.1	336	112	Cellular process	0.76
WP_013747282.1	339	113	Information and storage	1.18
WP_013746197.1	357	119	Cellular process	1.17
WP_013747255.1	366	122	Metabolism molecule	1.68
WP_013745138.1	375	125	Cellular process	1.11
WP_013745022.1	378	126	Cellular process	0.99
WP_013745048.1	381	127	Metabolism molecule	1.84
WP_043885299.1	381	127	Information and storage	0.67
WP_000460651.1	387	129	Cellular process	0.52
WP_013745244.1	396	132	Cellular process	1.17
WP_013746684.1	402	134	Cellular process	1.36
WP_013747139.1	405	135	Information and storage	0.98
WP_013744860.1	423	141	Metabolism molecule	0.48
WP_013745195.1	423	141	Metabolism molecule	0.77
WP_013747005.1	435	145	Metabolism molecule	1.18
WP_013745158.1	438	146	Cellular process	1.06
WP_013745185.1	444	148	Cellular process	1.74
WP_013745642.1	447	149	Metabolism molecule	1.29
WP_013745028.1	453	151	Cellular process	2.14
WP_013745278.1	453	151	Metabolism molecule	1.89
WP_013747134.1	462	154	Cellular process	1.39
WP_013747286.1	468	156	Cellular process	0.87
WP_013747276.1	471	157	Cellular process	1.4
WP_013747281.1	474	158	Cellular process	0.86
WP_013745799.1	480	160	Cellular process	0.89
WP_148234041.1	486	162	Metabolism molecule	1.33
WP_013745312.1	492	164	Cellular process	1.41
WP_013745886.1	492	164	Virulence factor	0.78
WP_026211100.1	492	164	Cellular process	1.14
WP_013746431.1	495	165	Metabolism molecule	1.41
WP_013744978.1	507	169	Cellular process	0.99
WP_013745596.1	507	169	Cellular process	0.65
WP_013745845.1	507	169	Cellular process	0.89
WP_013745596.1	507	169	Cellular process	0.69
WP_013745596.1	507	169	Cellular process	0.76
WP_013746870.1	510	170	Cellular process	0.98
WP_013745329.1	519	173	Virulence factor	0.83

WP_013747262.1	522	174	Metabolism molecule	1.33
WP_013746279.1	531	177	Cellular process	0.69
WP_013744944.1	555	185	Metabolism molecule	2.38
WP_013746187.1	555	185	Virulence factor	2.92
WP_013746280.1	555	185	Cellular process	1.24
WP_013745174.1	558	186	Cellular process	0.86
WP_013746743.1	561	187	Metabolism molecule	1.47
WP_013746394.1	567	189	Cellular process	1.53
WP_013746844.1	567	189	Cellular process	1.43
WP_013745016.1	573	191	Cellular process	1.15
WP_013745850.1	585	195	Metabolism molecule	0.63
WP_013747270.1	585	195	Information and storage	0.99
WP_013747209.1	588	196	Cellular process	0.85
WP_013745921.1	594	198	Metabolism molecule	1.34
WP_013746706.1	600	200	Cellular process	0.65
WP_013747010.1	609	203	Cellular process	1.13
WP_013746704.1	618	206	Cellular process	1.43
WP_148234044.1	624	208	Metabolism molecule	1.54
WP_013746702.1	624	208	Cellular process	0.88
WP_013746184.1	639	213	Cellular process	0.73
WP_013745528.1	648	216	Metabolism molecule	1.24
WP_013747164.1	654	218	Cellular process	0.93
WP_013746430.1	657	219	Cellular process	0.93
WP_080558815.1	663	221	Information and storage	0.34
WP_013747133.1	681	227	Cellular process	1.15
WP_013745598.1	711	237	Virulence factor	1.05
WP_013745266.1	732	244	Cellular process	1.55
WP_013745526.1	732	244	Cellular process	1.67
WP_013746703.1	738	246	Metabolism molecule	1.24
WP_013746625.1	741	247	Metabolism molecule	1.09
WP_013744870.1	747	249	Cellular process	0.49
WP_013745318.1	747	249	Cellular process	1.03
WP_013747269.1	753	251	Virulence factor	0.45
WP_013746790.1	762	254	Cellular process	1.63
WP_039155074.1	795	265	Metabolism molecule	1.89
WP_043885246.1	795	265	Metabolism molecule	1.8
WP_043885248.1	795	265	Metabolism molecule	1.22
WP_039155074.1	795	265	Metabolism molecule	1.23
WP_013747129.1	798	266	Cellular process	1.69
WP_013746869.1	804	268	Metabolism molecule	2.01
WP_013746281.1	819	273	Cellular process	1.59
WP_013745968.1	825	275	Metabolism molecule	1.21
WP_148234055.1	825	275	Metabolism molecule	0.67

WP_013745861.1	831	277	Virulence factor	0.73
WP_013745145.1	834	278	Cellular process	0.96
WP_013745467.1	834	278	Metabolism molecule	1.51
WP_013746168.1	834	278	Cellular process	0.87
WP_013745806.1	867	289	Metabolism molecule	1.15
WP_013745189.1	870	290	Metabolism molecule	1.77
WP_013746347.1	912	304	Metabolism molecule	0.98
WP_013746410.1	930	310	Cellular process	0.76
WP_013746557.1	942	314	Cellular process	1.55
WP_013746253.1	1,023	341	Metabolism molecule	2.45
WP_013745466.1	1,056	352	Cellular process	0.55
WP_013746421.1	1,062	354	Cellular process	1.22
WP_013746409.1	1,074	358	Metabolism molecule	0.89
WP_013745346.1	1,110	370	Virulence factor	1.22
WP_013746977.1	1,116	372	Virulence factor	1.03
WP_013746252.1	1,125	375	Cellular process	0.59
WP_013745398.1	1,137	379	Cellular process	1.89
WP_013745507.1	1,140	380	Cellular process	1.03
WP_013745468.1	1,155	385	Cellular process	1.48
WP_013746186.1	1,155	385	Cellular process	0.89
WP_013745235.1	1,182	394	Metabolism molecule	0.67
WP_013747156.1	1,212	404	Metabolism molecule	2.41
WP_013746705.1	1,260	420	Virulence factor	2.53
WP_013745126.1	1,263	421	Metabolism molecule	3.5
WP_013747206.1	1,263	421	Cellular process	1.51
WP_013745253.1	1,266	422	Cellular process	1.23
WP_013747057.1	1,329	443	Cellular process	1.93
WP_013746251.1	1,341	447	Cellular process	1.19
WP_013745202.1	1,350	450	Cellular process	1.88
WP_013746639.1	1,356	452	Cellular process	0.89
WP_013747271.1	1,602	534	Metabolism molecule	1.56
WP_043885272.1	3,033	1,011	Virulence factor	1.15
WP_013745844.1	5,241	1,747	Information and storage	0.89
