# Supplementary material for: In silico detection and characterization of novel virulence proteins of the emerging poultry pathogen Gallibacterium anatis
Source: Genomics Inform. 2022 Dec 30;20(4):e41. doi: 10.5808/gi.22006 (PMC9847380; doi:10.5808/gi.22006)
Supplement: Supplementary Table. 2. — Novel proteins identified from poultry pathogen Gallibacterium anatis associated with virulence, metabolism, Information and cellurer processes. [file gi-22006suppl2.pdf]

Supplementary Table 2. ?P

Factor	Novel proteins
Virulence factor	11
Metabolism molecule	61
Information and storage	10
Cellular process	119
