# Supplementary material for: In silico detection and characterization of novel virulence proteins of the emerging poultry pathogen Gallibacterium anatis
Source: Genomics Inform. 2022 Dec 30;20(4):e41. doi: 10.5808/gi.22006 (PMC9847380; doi:10.5808/gi.22006)
Supplement: Supplementary Table. 3. — Functional domain prediction by five web servers for the virulence protein WP_013745346.1 [file gi-22006suppl3.pdf]

Supplementary Table 3. Functional domain prediction by five web servers for the virulence protein WP_013745346.1

Family id	Env. Start	Env. End
Citrate_synthase	3	351
Citrate_synthase	1	369
Citrate_synthase	3	351
Citrate_synthase	1	369
Citrate_synthase	3	351
