# Supplementary material for: In silico detection and characterization of novel virulence proteins of the emerging poultry pathogen Gallibacterium anatis
Source: Genomics Inform. 2022 Dec 30;20(4):e41. doi: 10.5808/gi.22006 (PMC9847380; doi:10.5808/gi.22006)
Supplement: Supplementary Fig. 1. — Phylogenetic relationships of the virulence protein WP_013745346.1 with other reference protein sequences. (The evolutionary history was inferred using the neighbor-joining method. The bootstrap tree was inferred from 1,000 replicates and taken to represent the evolutionary history. The evolutionary distances were computed using the Poisson correction method and are in the units of the number of amino acid substitutions per site. The analysis involved 15 amino acid sequences. There was a total of 262 positions in the final dataset. The evolutionary analysis was conducted in MEGA7). [file gi-22006suppl4.pdf]

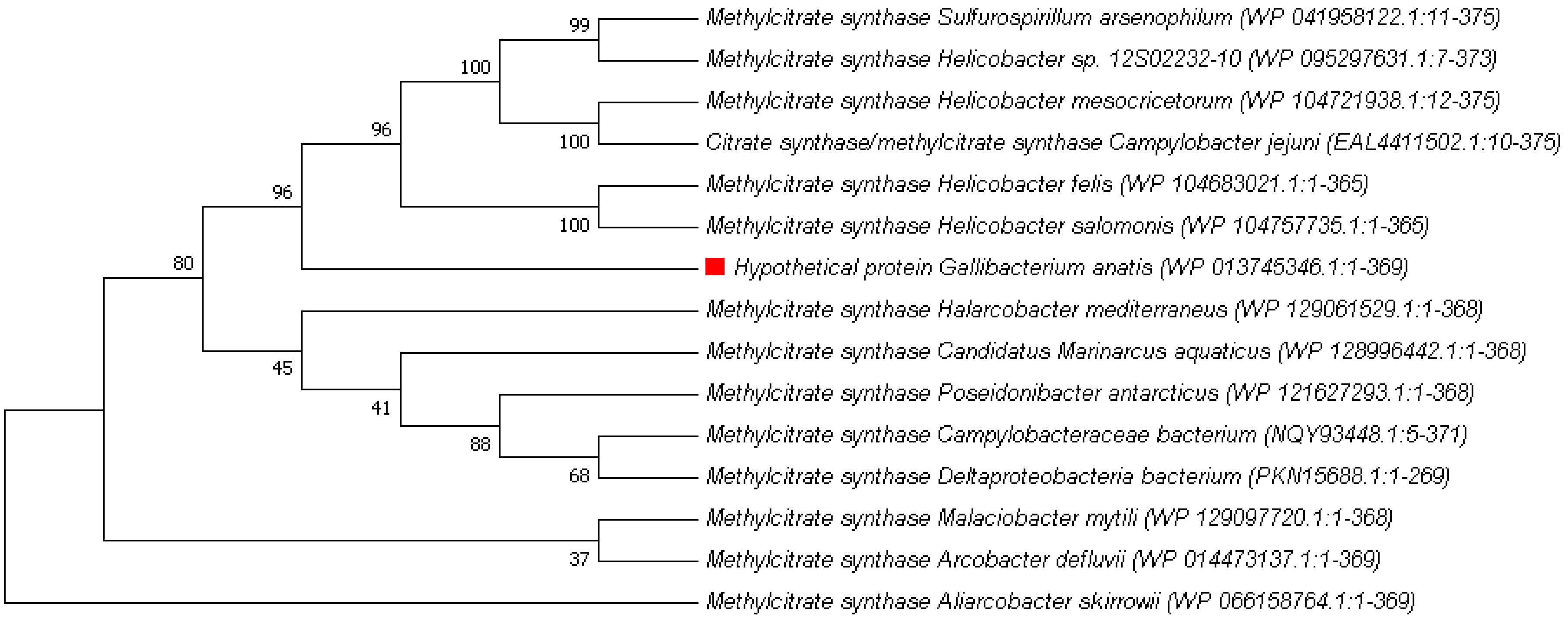

Supplementary Fig. 1. Phylogenetic relationships of the virulence protein WP_013745346.1 with other reference protein sequences. (The evolutionary history was inferred using the neighbor-joining method. The bootstrap tree was inferred from 1,000 replicates and taken to represent the evolutionary history. The evolutionary distances were computed using the Poisson correction method and are in the units of the number of amino acid substitutions per site. The analysis involved 15 amino acid sequences. There was a total of 262 positions in the final dataset. The evolutionary analysis was conducted in MEGA7).
