# Supplementary figures and images for: In silico detection and characterization of novel virulence proteins of the emerging poultry pathogen Gallibacterium anatis
Source: Genomics Inform. 2022 Dec 30;20(4):e41. doi: 10.5808/gi.22006 (PMC9847380; doi:10.5808/gi.22006)

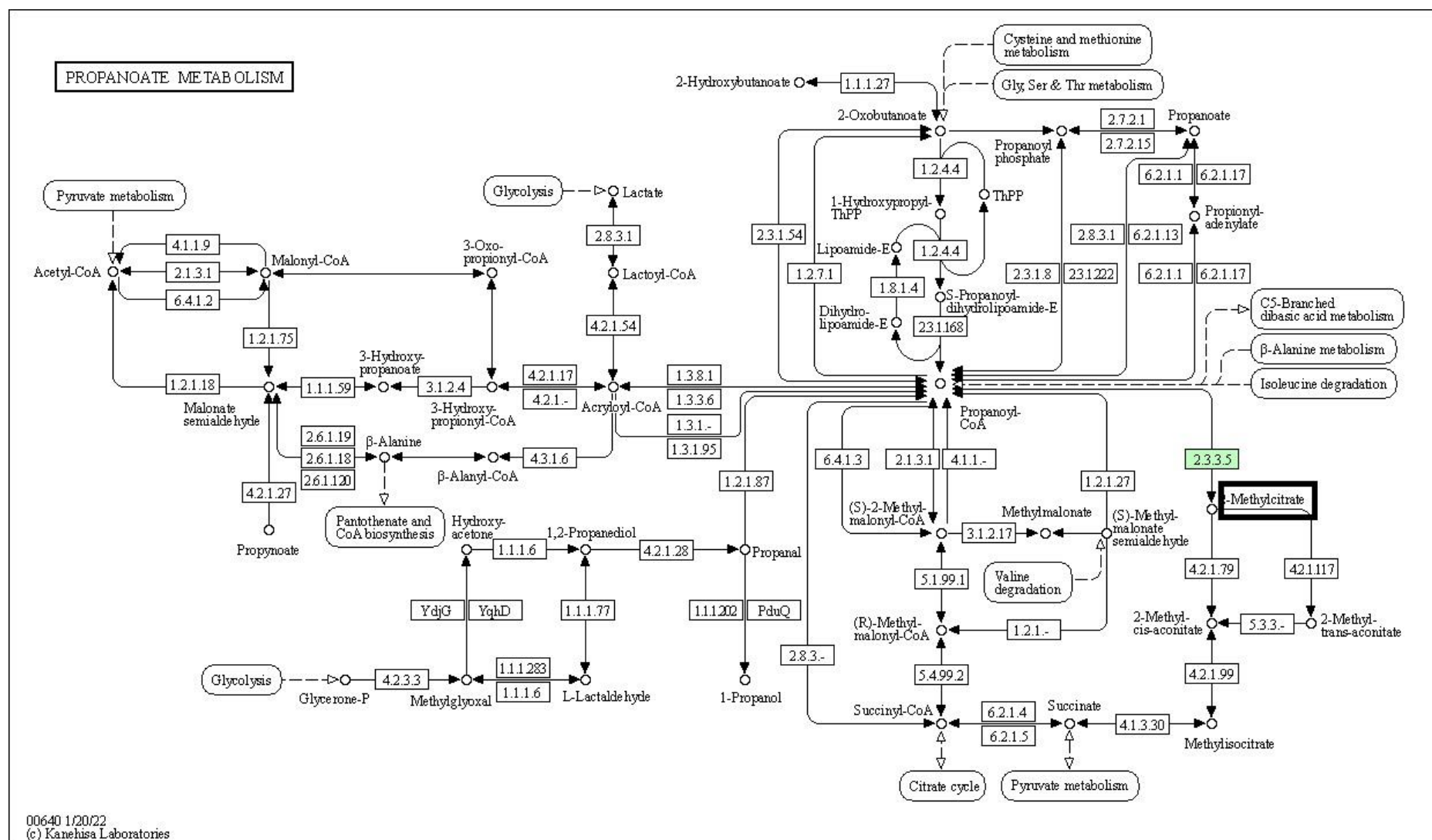

Supplement: Supplementary Fig. 2. — Potential metabolic pathways associated with the virulence protein WP_013745346.1 by KAAS (KEGG automatic annotation server analysis). [file gi-22006suppl5.pdf]
