# Supplementary material for: In silico detection and characterization of novel virulence proteins of the emerging poultry pathogen Gallibacterium anatis
Source: Genomics Inform. 2022 Dec 30;20(4):e41. doi: 10.5808/gi.22006 (PMC9847380; doi:10.5808/gi.22006)
Supplement: Supplementary Fig. 3. — Comparative analysis of the 3D structure, structural domains, cellular location, and molecular functions of reference methylcitrate synthases to WP_013745346.1 [file gi-22006suppl6.pdf]

Name	3D structure	Functional domain	Cellular location	Molecular function
C. burnetii	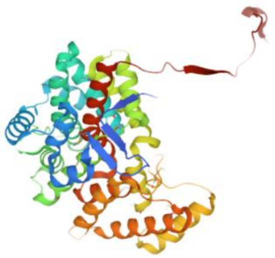	Citrate synthase, C-terminal domain	Cytoplasm	Catalytic activity, transferase activity, acyltransferase activity, acyl groups converted into alkyl on transfer, 2-methylcitrate synthase activity, citrate (Si)-synthase activity, citrate synthase activity, lyase activity, carbon-carbon lyase activity, oxo-acid-lyase activity
M. tuberculosis	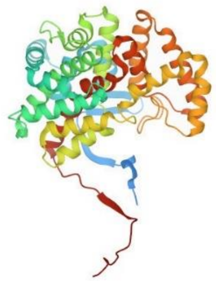	Citrate synthase, C-terminal domain	Cytoplasm	Catalytic activity, transferase activity, acyltransferase activity, acyl groups converted into alkyl on transfer, 2-methylcitrate synthase activity, citrate (Si)-synthase activity, citrate synthase activity, lyase activity, carbon-carbon lyase activity, oxo-acid-lyase activity
G. anatis WP_013745346.1	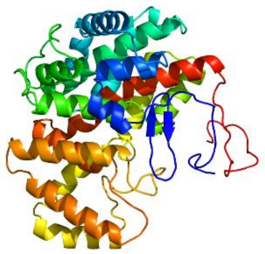	Citrate synthase, C-terminal domain	Cytoplasm	Catalytic activity, transferase activity, acyltransferase activity, acyl groups converted into alkyl on transfer, 2-methylcitrate synthase activity, citrate (Si)-synthase activity, citrate synthase activity, lyase activity, carbon-carbon lyase activity, oxo-acid-lyase activity

Supplementary Fig. 3. Comparative analysis of the 3D structure, structural domains, cellular location, and molecular functions of reference methylcitrate synthases to WP_013745346.1
